# An exploratory, in‐field, multimodal assessment of cervical function in adults following to high‐altitude trekking with light backpack

**DOI:** 10.14814/phy2.71060

**Published:** 2026-08-18

**Authors:** David Perpetuini, Vittore Verratti, Danilo Bondi, Arcangelo Merla, Egidio Botta, Michele D'Attilio

**Affiliations:** ^1^ BioEng Lab, Department of Engineering and Geology University G. d'Annunzio of Chieti‐Pescara Pescara Italy; ^2^ Department of Science University “G. d'Annunzio” Chieti‐Pescara Chieti Italy; ^3^ Italian Society of Mountain Medicine Padova Italy; ^4^ Department of Neuroscience, Imaging and Clinical Sciences University “G. d'Annunzio” Chieti‐Pescara Chieti Italy; ^5^ Department of Life, Health and Environmental Sciences University of L'aquila L'Aquila Italy; ^6^ Department of Innovative Technologies in Medicine & Dentistry University “G. D'annunzio” Chieti‐Pescara Chieti Italy

**Keywords:** backpack, head and neck, inertial measurement unit, range of motion, surface electromyography, thermal imaging, trapezius

## Abstract

Hours‐lasting walks with backpacks can trigger or worsen musculoskeletal dysfunctions of the neck, and high‐altitude hypoxia adds to the stress. To delve deeper into this topic, this study analyzed the functional and neuromuscular alterations at the head‐and‐neck region during high‐altitude trekking by combining thermal imaging, electromyography, and kinematics. Fifteen adults who took part in a Himalayan expedition were tested for cervical range of motion (RoM) at 1400 m, at peak altitude staying (5000 meters), and once back at 1400 m, during a trek lasting 14 days. The 8 males were also tested for surface electromyography (sEMG) and infrared thermography. The maximum RoM of flexion‐extension along the sagittal plane increased in response to trekking. Surface electromyography revealed greater activation at the end of the trek in right side for rotation and the left side for bending. Thermography revealed a general reduction of skin temperature over the target muscles during side bending. This study highlights the feasibility and usefulness of multimodal analysis through inertial measurement unit‐registered kinematics, thermography, and electromyography in monitoring neck function in a demanding real‐world environment. The high‐altitude trek, with a backpack weighing less than 10% of one's body weight, triggers asymmetrical adaptations in the neck muscles, increasing measured RoM.

## Graphical abstract

This study investigates the impact of a high‐altitude expedition (1400 m to 5000 m) on cervical spine dynamics using an integrated approach: kinematics (IMU), surface electromyography (sEMG), and infrared (IR) thermography. The findings highlight a progressive increase in cervical range of motion (RoM) during flexion‐extension and lateral tilting. Additionally, the protocol reveals task‐specific asymmetrical muscle adaptations in the trapezius and differentiated thermal responses, notably a general reduction in skin temperature during lateral tilting.

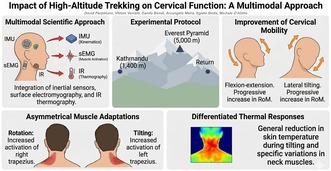

## 1 INTRODUCTION

A variety of typical actions interfere with head and neck biomechanical patterns; among these, backpack carrying is one of the most studied (Ellapen et al., 2021). This is a daily activity for many students and workers and stands for the prototypical representation of trekkers and hikers. The backpack's heft and design have a significant impact on posture and biomechanics. In order to avoid pain, postural alterations, and perceived exertion, lower the risk of lower limb injuries and minimize strain on the neck and shoulders, it is recommended that hikers impose a load restriction of 30% of body weight, while positioning the backpack high on the back and keeping the shoulder straps short (Abdelraouf et al., 2016; Simpson et al., 2011a, 2011b). Excessive and/or non‐symmetric load of a backpack may result in musculoskeletal aberrant functions, thus leading to a loss of physiological range of motion.

Mandibular and body posture are interconnected due to anatomical and biomechanical factors. Reduced intraoral negative pressure increases the activity of elevating muscles, compensating with increased postural tone. Postural orientation is essential for stability due to intrinsic instability. Movements continuously change body posture, while mandible posture must remain constant to avoid damaging dental arches and oral cavity soft tissues. There is scientific disagreement regarding the connection between posture and occlusion, and the literature presents conflicting findings (e.g. Bracco et al., 2004; Perinetti, 2006). According to a recent review, there appears to be a relationship between dental occlusion and posture, but only in the presence of outside disruptions including tasks to be completed, weariness, and unstable support surfaces (Julià‐Sánchez et al., 2019). The complexity of this relationship may depend on the biomechanical chains involved in the head and neck district's functions, and additional elements like macrotraumas, neuroendocrine stress axis activations, and muscular parafunctions have long been known (Stohler, 1999; Zhang et al., 1999). Evidence demonstrates age‐related decline in cervical mobility, albeit in a non‐continuous trend (Pan et al., 2018). Unilateral posterior neck pain also affects the cervical district, as patients exhibit greater asymmetry of neck motion and muscle activation during prone neck extension (Park et al., 2017). Acute functional compensations may result from the above‐mentioned functional limitations, and if they persist over time, structural compensations may follow.

Within this background, during high‐altitude expeditions the trekkers are often warned about possible cervical dysfunctions. Hours‐lasting walks while carrying backpacks can indeed trigger or worsen musculoskeletal dysfunctions of the neck, primarily through impairments in postural control and neuromuscular fatigability; the hypoxic stress associated with high‐altitude walks can further overstress physiological systems, thereby indirectly affecting neck pain, stability, and function.

Thermal imaging is considered a viable tool for monitoring human thermal responses to hypoxia (Jones et al., 2018) and it is widely used to assess thermal distribution, asymmetries, tension, and activation in head and neck muscles (de Almeida et al., 2022). However, no study has used it to study possible alterations due to trekking at altitude hypoxia. Moreover, combining this analysis with kinematics and surface electromyography provides an optimal view of muscle activation and its effects on movements in the head and neck area by using non‐invasive and portable methods.

### 1.1 Aims of the study

The present study aimed to explore the functional and neuromuscular alterations at the head–neck district level during high‐altitude trekking by combining thermal imaging, electromyography, and range‐of‐motion analysis. This represented a novelty, as it constituted the first multimodal in‐field investigation on this specific topic.

## 2 MATERIALS AND METHODS

### 2.1 Study design

During the journey, measurements were taken at T0, before the start of the trek (Kathmandu, ≈1400 m a.s.l., atmospheric pressure of 656 mmHg), one measurement at T1 (Pyramid Laboratory, Everest National Park, ≈5000 meters, atmospheric pressure of 418 mmHg) after 6 days of trekking, and one at T2 upon returning to Kathmandu. The ascent trek towards the Pyramid Laboratory, starting from Lukla airport, took place over 6 days interspersed with a rest day in Namche Bazar. The descent trek, after 5 days of staying at the Pyramid, took place over 4 days. Phases of ascent and descent alternated, without the use of special equipment such as ice axes or crampons; the backpacks carried on the participants' back and shoulders generally had a light load, ranging from 3 to 10 kg, in all cases lower than 10% of body weight. All study procedures performed on the expedition members were conducted in accordance with the ethical standards of the 1964 Declaration of Helsinki and its subsequent amendments. This work constitutes an ancillary element of a study approved by the local Institutional Review Board (‘Comitato Etico delle Province di Chieti e Pescara’, document no. 18, 29/07/2021) on hypoxia (Bondi et al., 2025, 2026) and integrates previous results on motor functions at high altitude (D'Attilio et al., 2026). Written informed consent was obtained from each of the study participants.

### 2.2 Participants

The present study was conducted on a sample of 15 volunteers (8 males and 7 females) with a mean age of 41.7 ± 14.7 years and a mean BMI of 23.9 ± 3.46 kg/m^2^, who ascended to high altitude in the Himalayan Mountain range. The exclusion criteria were:

- ongoing ischemic heart disease
- previous acute myocardial infarction (AMI)
- chronic obstructive pulmonary disease (COPD)
- psychosis, neurosis, schizophrenia, depression, neurological diseases
- alcoholism, drug addiction
- respiratory failure
- uncontrolled hypertension
- anticoagulant therapy

All participants exhibited no symptoms indicative of unilateral or central posterior neck pain. None of them used walking poles regularly while trekking. None of them took analgesic drugs during the testing days. Oxyhemoglobin saturation assessed via arterial blood gas analysis ranged from 95% ± 2% at T0 to 77% ± 8% at T1 and 96% ± 1% at T2.

For range‐of‐motion (RoM) analysis all 15 participants were included, while surface electromyography (sEMG) and infrared (IR) thermography were conducted on males only (age of 42.4 ± 13.4 years and a BMI of 25.4 ± 2.96 kg/m^2^).

### 2.3 Procedures

#### 2.3.1 Range‐of‐motion (RoM) recording and analysis

The cervical RoM was assessed in the three planes by asking the volunteer to perform right and left rotation on transversal plane, flexion and extension on thesagittal plane, right and left flexion on thecoronal plane (side bending), up to the maximum RoM achievable, without the speed constraint. The test was conducted by using Baiobit (Rivelo, BTS Bioengineering Group, Italy), a lightweight sensor (70 × 40 × 18 mm; weight ≈37 g), whose inertial measurement unit (IMU) includes three‐dimensional gyroscope, accelerometer, and magnetometer; its reliability and validity for assessing cervical movements were previously demonstrated (Nota, Pittari, Gamba, et al., 2023; Wang et al., 2024). The instrument was placed on the external occipital protuberance by an adjustable band attached to the head (Palmieri et al., 2023). Participants were asked to avoid trunk displacement, which was supervised by the experimenter. In addition to maximal RoM, the mean angular velocity during each movement was also collected; however, considering the time to reach max RoM was not a constraint, that variable was not further analyzed.

#### 2.3.2 Surface electromyography (sEMG) recording and analysis

Surface electromyography (sEMG) was recorded with Due+ (OT Bioelettronica, Torino, Italy). Raw sEMG from the two channels were registered in bipolar configuration with a sampling frequency of 2048 Hz, filtered with bandpass 10–500 Hz, and converted to digital data. Signals were recorded and further visualized with the software OT Biolab+ v1.5.6 (OT Bioelettronica). The skin was prepared by abrasion and cleaning and the two pairs ad adhesive electrodes were placed on the upper trapezius. Positioning was consistent between each condition and participant. The Surface ElectroMyography for the Non‐Invasive Assessment of Muscles (SENIAM) guidelines for sensor locations were not strictly followed due to the concurrent infrared thermography recording. Specifically, following manual palpation of the C7 spinous process, the lower electrode was placed over the trapezius muscle belly approximately at the level of an imaginary horizontal line through C7. The upper electrode was positioned along the muscle belly, with its lower edge maintained at an inter‐electrode distance of approximately 2 cm from the upper edge of the lower electrode. The quality of sEMG signals was checked before the registration by estimating resting RMS to evaluate the noise level (to be lower than 10 μV).

The sEMG raw signals were rectified, and the amplitude of muscle activity was subsequently quantified using RMS over non‐overlapping windows of 250 ms. Concurrently, the integrated EMG (iEMG) was determined by calculating the sum of the rectified signal in the same windows. Both parameters were normalized to the maximum over tasks and subjects for between‐subject comparison.

#### 2.3.3 Infrared (IR) thermography recording and analysis

The FLIR SC660 digital thermal infrared camera (FLIR, Wilsonville, OR, USA, 640 × 480 bolometer FPA, sensitivity/noise equivalent temperature difference of 30 mK at 30°C, and field of view of 24° by 18°) was used to assess skin temperature. The camera was positioned 60 cm from the participants, directed at their backs, concentrating on the top section, including the neck. The frequency rate was set at 10 Hz. The procedure was set in accordance with guidelines (Diakides et al., 2012; Merla & Romani, 2005; Ring & Ammer, 2012): the camera underwent blackbody calibration to mitigate any sensor response drift and optical artifacts; the participants entered the experimental room 15 min before the session's beginning to enable the stabilization of their baseline skin temperature; the room's temperature and relative humidity were approximately at 22°C–25°C and 30%–50%, respectively, although there was no thermostat for precise regulation.

**FIGURE 1:**
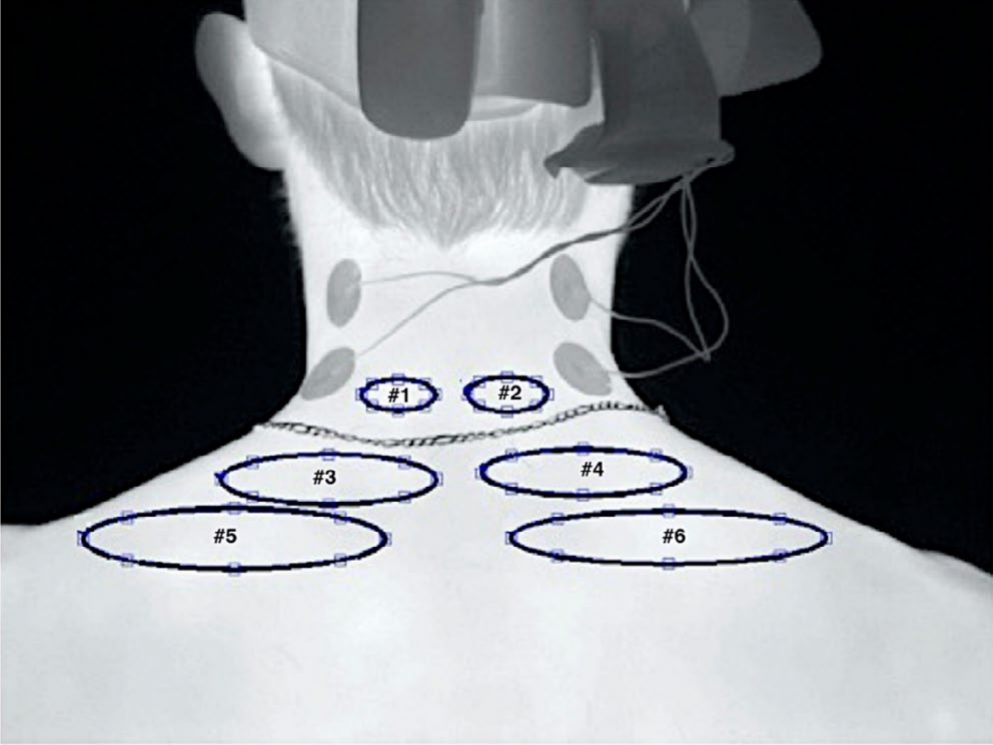
ROIs placement on a representative participant, who provided written informed consent for publishing this image.

Regarding the data analysis, a first evaluation of the collected thermal signals' quality was conducted by visual inspection, and no video was discarded. Six regions of interest (ROI) were selected on the neck and upper back, as seen in Figure 1. The locations of these ROIs were monitored throughout all frames of the thermal video using an automatic tracking algorithm (Perpetuini et al., 2021). The thermal time course was normalized by subtraction of the first frame, and the average value of temperature for each ROI was assessed.

Notably, sEMG, kinematic assessments, and thermal imaging measurements were performed in the same session and concurrently; therefore, all measurements were conducted in acontrolled setting, thus mitigating the extreme temperature fluctuations of the external high‐altitude environment that could impact the reliability of the IMU measurements.

#### 2.3.4 Statistical comparisons

Prior to carrying out statistical comparisons between the multiple study times and the two sides, normality of residuals was checked through assumption test coupled with Q‐Q plots observation. Then, basing on distribution observed and possible outlier removal (through an iterative process involving the removal of data points exceeding two standard deviations from the mean, checking normality, and repeating this process until the assumptions were met), a series of repeated measures‐analyses were carried out by setting both side and altitude as within factors; the Greenhouse–Geisser correction was applied when the assumption of sphericity was violated, as assessed by Mauchly's test, and post‐hoc tests were carried out with Holm‐Šídák's method for multiple comparison. All analyses and graphs were conducted with Prism Version 10.4.0 (GraphPad Software, San Diego, USA). The effect size was estimated as partial eta squared (*η* ^2^_p_), calculated from the F statistics as *η* ^*2*^_p_ *= (F × df1)/(F × df1 + df2)*, and reported together with the 95% confidence interval.

## 3 RESULTS

### 3.1 RoM

**FIGURE 2:**
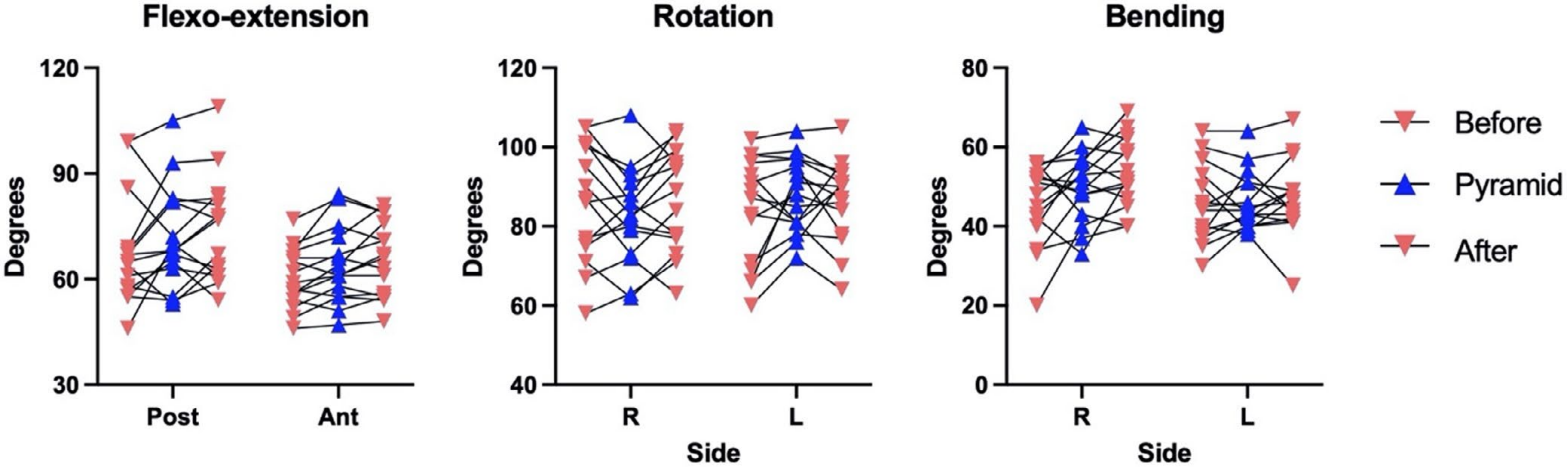
Results of cervical spine movements' range of motion, in anterior and posterior flexion (flexo‐extension), right–left rotation (rotation), and side bending (bending).

Regarding head RoM, there were no statistically appreciable changes in rotation by altitude, while there were increases both in antero‐posterior flexion‐extension (*p* = 0.001, *F*_(2,28)_ = 9.434, *η* ^2^_p_ = 0.403 [95% CI 0.100, 0.576]) and in bending (*p* = 0.048, *F*_(1.67,23.39)_ = 3.678, *η* ^2^_p_ = 0.208 [95% CI 0.000, 0.431]); in each case, increases tended to be progressive during the study timeline. Both for rotation and bending, there were differential trends across sides during the study timeline (side × altitude: *p* = 0.001, *F*_(1.42,19.87)_ = 6.983, *η* ^2^_p_ = 0.333 [95% CI 0.026, 0.553] for rotation; *p* = 0.023, *F*_(1.95,27.35)_ = 4.407, *η* ^2^_p_ = 0.239 [95% CI 0.002, 0.442] for bending); in particular, the right flexion exhibited the most appreciable increase once back in Kathmandu (before‐after, *p*_adjusted_ = 0.025), as shown in Figure 2.

The analysis on males only confirmed the increases in antero‐posterior flexion‐extension (*p* = 0.034, *F*_(2,14)_ = 4.343, *η* ^2^_p_ = 0.383 [95% CI 0.000, 0.598]) across study times, but not that on bending. There remained differences, although non statistically significant, on side × altitude differences for bending (*p* = 0.085, *F*_(2,14)_ = 2.955, *η* ^2^_p_ = 0.297 [95% CI 0.000, 0.533]), but not for rotation.

### 3.2 sEMG

**FIGURE 3:**
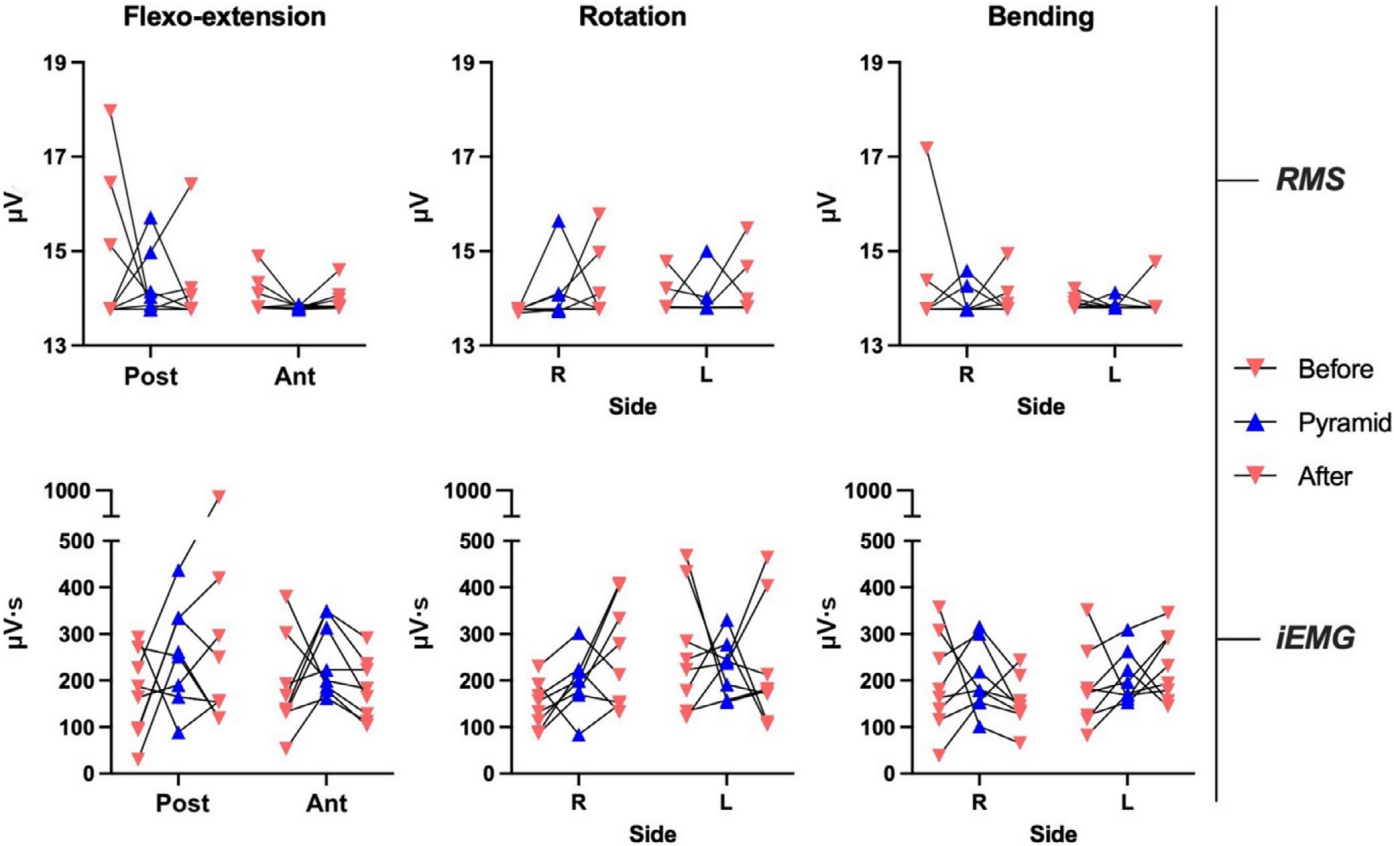
Results of root mean square (RMS) and integrated electromyography (iEMG) during anterior and posterior cervical flexion (flexo‐extension), right–left cervical rotation (rotation), and cervical side bending (bending).

Regarding RMS, there were no statistically appreciable changes in rotation, flexo‐extension and bending by altitude, nor in the side × altitude comparisons. Regarding iEMG, there were no statistically appreciable changes in rotation, flexo‐extension and bending by altitude; both for rotation and bending, there were differential trends across sides during the study timeline (side × altitude: *p* = 0.042, *F*_(1.69,11.80)_ = 4.439, *η* ^2^_p_ = 0.389 [95% CI 0.000, 0.619] for rotation; *p* = 0.088, *F*_(1.84,12.90)_ = 3.008, *η* ^2^_p_ = 0.300 [95% CI 0.000, 0.546] for bending), with increased value once back in Kathmandu in right side for rotation and left side for bending, as shown in Figure 3.

### 3.3 IR imaging

**FIGURE 4:**
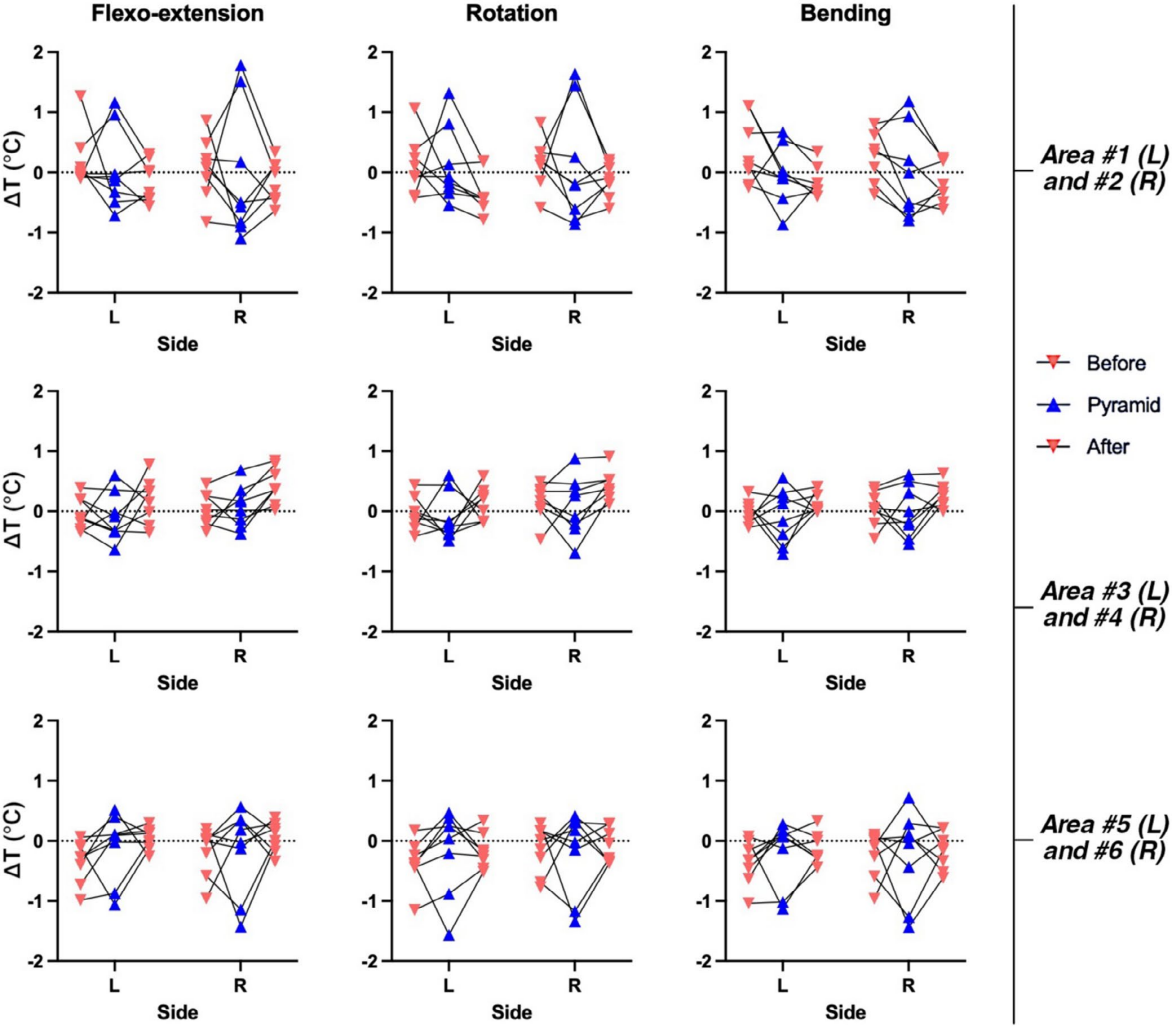
Results of thermal imaging during anterior and posterior cervical flexion (flexion‐extension), right–left cervical rotation (rotation), and cervical side bending (bending).

Regarding areas #1 and #2, the most consistent result regards the decrease in mean temperature during bending once back in Kathmandu (*p* = 0.033, *F*_(1.88,13.15)_ = 4.559, *η* ^2^_*p*_ = 0.395 [95% CI 0.000, 0.612]). Regarding areas #3 and #4, as shown in Figure 4, increased values seem to emerge at the end of the expedition over pre‐expedition values, particularly for flexo‐extension (*p* = 0.054, *F*_(2,14)_ = 3.637, *η* ^2^_p_ = 0.342 [95% CI 0.000, 0.567]) in the right side (before‐after, *p*_adjusted_ = 0.039; Pyramid‐after, *p*_adjusted_ = 0.022). For what concerns the areas #5 and #6, there were no statistically appreciable changes in rotation, flexo‐extension and bending by altitude, nor by side × altitude.

## 4 DISCUSSION

Humans' muscle activity and gait patterns can be affected by walking with external loads that are over 10% of body weight, but lighter loads may be even beneficial by reducing lumbosacral compression forces (Li & Chow, 2018; Matur et al., 2025). Along the same lines, our results show that high‐altitude trekking with light loads leads to a slight increase in maximum cervical RoM. The increase in cervical antero‐posterior RoM is of note, as the most notable difference in cervical RoM of patients with temporo‐mandibular disorders regards reduced amplitude of cervical extension (Nota, Pittari, Lannes, et al., 2023). As regards muscle status, differences were observed in terms of increased electromyographic activation of the two sides during rotation and side bending, with a general reduction in activation during anterior–posterior flexion‐extension tasks. Thermography, on the other hand, showed a general reduction of skin temperature during side bending, with a different temperature increase between the two sides during anteroposterior flexion‐extension tasks. However, it is worth highlighting that skin temperature is an indirect measure of muscle activity; thus, the temperature changes observed may be influenced by localized vascular alterations, changes in skin perfusion due to altitude‐induced hypoxia, or minor environmental fluctuations, rather than muscle activation alone.

**FIGURE 5:**
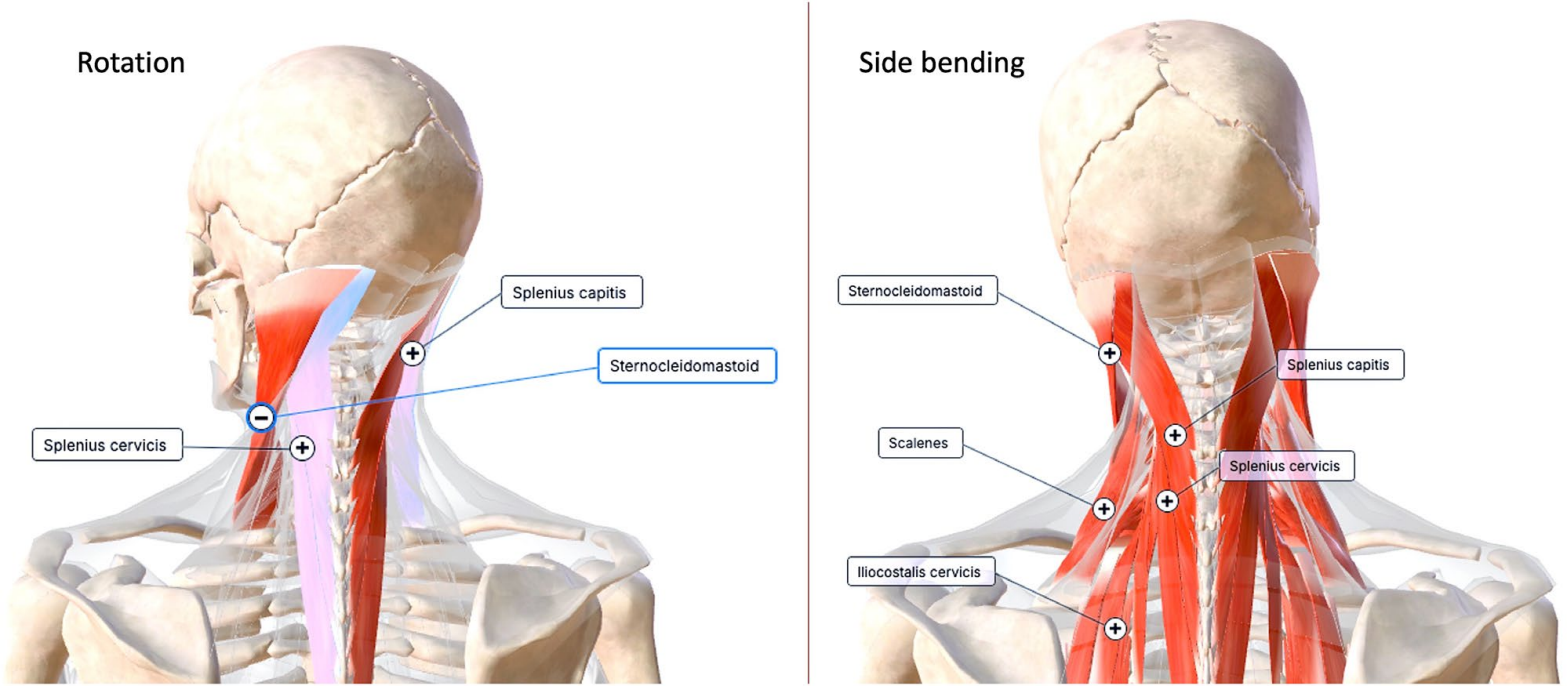
Main muscles involved in cervical rotation and side bending. Image was created with The BioDigital Human interactive atlas (human.biodigital.com/explore).

The muscles involved in the rotation, flexo‐extension, and bending of the head include the sternocleidomastoid, trapezius, and erector spinae. In particular, (1) muscles responsible for anterior flexion are the sternocleidomastoid, longus colli, longus capitis, and the anterior scalene, while the posterior flexion is mainly due to splenius capitis, with anadditional role of splenius cervicis, semispinalis capitis, the upper fibers of the trapezius, and the erector spinae; (2) rotation is powered mainly by the contralateral sternocleidomastoid, with the secondary role due to ipsilateral splenius muscles and a minor role of the contralateral upper trapezius; (3) side bending is due to the ipsilateral anterior, middle, and posterior scalenes, with supporting role of ipsilateral sternocleidomastoid, levator scapulae, erector spinae, upper trapezius and splenius muscles (see Figure 5).

The fact that differential effects were observed by side in EMG and thermal imaging reflects the ipsilateral and contralateral activation of different muscles during cervical rotation and side bending. Moreover, the placement of EMG was set on the upper trapezius, which usually plays a minor role in head movement, but creates a strong clavicular elevation (Camargo & Neumann, 2019) and its altered stiffness is associated with neck pain with no necessary limitations on head movements (Seidel et al., 2023). The altitude trek with light backpack has slightly altered the head and neck biomechanics, increasing the contribution of the right side during rotation and left side during side bending. Thermal imaging, which indeed did not focus on the upper trapezius but on the lower muscles, depicted a relatively greater activation of secondary muscles during backward extension and ipsilateral muscles during bending. This greater activation possibly aligns with the increased RoM of right bending. Nevertheless, it is not possible to infer a uniform model of muscle activation and deactivation during cervical movements, due to the inter‐individual heterogeneity in the impact of altitude trek on the muscles studied.

### 4.1 Limitations and perspectives

Firstly, the study sample was relatively small; therefore, future studies with a larger sample size are warranted; however, the design study is quite peculiar, thus enrolling further participants could be challenging. Despite the large effect sizes observed, limited small samples also can yield upwardly biased estimates of effect magnitude, actually reflected in the relatively wide 95% CI of the values, suggesting that the true effects may differ substantially from the observed estimates. The present study does not allow to distinguish between the relative contributions of trekking activity and high‐altitude hypoxia to the observed alterations in cervical function. The absence of a normoxic control group limits the ability to isolate hypoxia as a single contributing factor and consequently the interpretation of our results. It is plausible that the increases in cervical range of motion and the mild neuromuscular asymmetries primarily reflect functional and biomechanical adaptations to prolonged locomotor activity with light loads, but we cannot exclude the role of hypoxia. The effects of accumulated walking exposure, recovery status and repeated testing cannot be excluded. Moreover, sEMG was acquired only from the upper trapezius, but multichannel acquisitions could better describe the physiological processes related to walking with external loads. Normalizing sEMG data to the maximum value across all tasks and participants has limited between‐participant comparisons and the interpretation of muscle activation and coordination profiles during head movements.

Furthermore, it is important to acknowledge the potential environmental effects on sensor performance. While indoor room temperatures were kept relatively stable, the standard laboratory calibration of the IMU and sEMG sensors may not fully account for the uncompensated effects of extreme atmospheric pressure drops or temperature fluctuations at high altitudes, which could theoretically influence sensor bias, skin‐electrode impedance, and overall measurement stability.

Moreover, it should be highlighted that although standard guidelines for thermography measurements were adopted, this technique remains highly vulnerable to environmental and physiological confounding. Hence, the skin temperature variations assessed cannot be exclusively attributed to underlying muscle activation, but they could be influenced by autonomic vascular responses, altered skin perfusion secondary to physical exertion and high‐altitude hypoxia, or minor microclimatic changes.

Despite these limitations, this study delivers in‐field, multimodal evidence that backpacking at altitude induces modifications in cervical kinematics and muscle activity, detectable through thermography, sEMG, and IMU. The overall scientific and practical value of this work still remains, as it was conducted within an ecological, real‐world model that closely mirrors the conditions experienced by thousands of trekkers and mountaineers at high altitude.

## 5 CONCLUSIONS

The present study reports a multimodal assessment of task‐ and side‐specific cervical adaptations in response to walking with external loads at altitude in the Himalayas. High‐altitude trekking with light loads does not reduce, rather increase, the measured RoM of cervical flexo‐extension, and peculiar and differential modifications in skin temperature and muscle activations were observed. The present findings hold direct translational relevance for preventive, clinical, and ergonomic recommendations in mountain environments. Future research should include additional an analogue normoxic trek, or additional hypoxic exposures, to disentangle the role of hypoxia from that of physical exercise on cervical biomechanics and muscle function. Further studies may explore the role of poles in altitude trekking, as pole walking is more locally stable than walking at cervical level, thus reducing the kinematic variability in the upper trunk (Zoffoli et al., 2017). In conclusion, this study highlights the feasibility and usefulness of a multimodal approach encompassing IMU, infrared thermography, and sEMG to monitor neck function in a demanding real‐world environment.

## AUTHOR CONTRIBUTIONS

**David Perpetuini:** Conceptualization; formal analysis; methodology; resources; visualization. **Vittore Verratti:** Funding acquisition; project administration; resources. **Danilo Bondi:** Conceptualization; formal analysis; funding acquisition; investigation; methodology; visualization. **Arcangelo Merla:** Resources; supervision. **Egidio Botta:** Formal analysis. **Michele D'Attilio:** Conceptualization; investigation; methodology; project administration; resources; supervision.

## FUNDING INFORMATION

DB was supported by Vivisol S.r.l. (Monza, Italy). The funders had no role in the design of the study, data collection, analyses, or interpretation of the data, writing of the manuscript, or in the decision to publish the results.

## DECLARATION OF GENERATIVE AI AND AI‐ASSISTED TECHNOLOGIES

Nothing to disclose.

## ETHICS STATEMENT

This work constitutes an ancillary element of a study approved by the local Institutional Review Board (‘Comitato Etico delle Province di Chieti e Pescara’, document no. 18, 29/07/2021) on hypoxia and integrates previous results on motor functions at high altitude. Written informed consent was obtained from each of the study participants.

## Data Availability

Data are available from the Corresponding Author upon request.
